# Exosomal delivery of therapeutic modulators through the blood–brain barrier; promise and pitfalls

**DOI:** 10.1186/s13578-021-00650-0

**Published:** 2021-07-22

**Authors:** Morteza Heidarzadeh, Yasemin Gürsoy-Özdemir, Mehmet Kaya, Aysan Eslami Abriz, Amir Zarebkohan, Reza Rahbarghazi, Emel Sokullu

**Affiliations:** 1 Koç University Research Center for Translational Medicine (KUTTAM), Rumeli Feneri, 34450 Sariyer, Istanbul Turkey; 2 Neurology Department, Koç University School of Medicine, Rumeli Feneri, 34450 Sariyer, Istanbul Turkey; 3 Physiology Department, Koç University School of Medicine, Rumeli Feneri, 34450 Sariyer, Istanbul Turkey; 4 Department of Medical Nanotechnology, Faculty of Advanced Medical Sciences, Tabriz University of Medical Sciences, Tabriz, Iran; 5 Stem Cell Research Center, Tabriz University of Medical Sciences, Tabriz, Iran; 6 Department of Applied Cell Sciences, Faculty of Advanced Medical Sciences, Tabriz University of Medical Sciences, Tabriz, Iran; 7 Biophysics Department, Koç University School of Medicine, Rumeli Feneri, 34450 Sariyer, Istanbul Turkey

**Keywords:** Exosomes, Blood–brain barrier, Delivery

## Abstract

Nowadays, a large population around the world, especially the elderly, suffers from neurological inflammatory and degenerative disorders/diseases. Current drug delivery strategies are facing different challenges because of the presence of the BBB, which limits the transport of various substances and cells to brain parenchyma. Additionally, the low rate of successful cell transplantation to the brain injury sites leads to efforts to find alternative therapies. Stem cell byproducts such as exosomes are touted as natural nano-drug carriers with 50–100 nm in diameter. These nano-sized particles could harbor and transfer a plethora of therapeutic agents and biological cargos to the brain. These nanoparticles would offer a solution to maintain paracrine cell-to-cell communications under healthy and inflammatory conditions. The main question is that the existence of the intact BBB could limit exosomal trafficking. Does BBB possess some molecular mechanisms that facilitate the exosomal delivery compared to the circulating cell? Although preliminary studies have shown that exosomes could cross the BBB, the exact molecular mechanism(s) beyond this phenomenon remains unclear. In this review, we tried to compile some facts about exosome delivery through the BBB and propose some mechanisms that regulate exosomal cross in pathological and physiological conditions.

## Background

EVs, mainly exosomes, are subsets of naturally occurring particles inside the cells with notable functions during physiological and pathological conditions [1]. Recent data revealed that exosomes facilitate paracrine cell-to-cell communication via the transfer of different biomolecules [2]. Evidence points to the fact that these nanoparticles can deliver numerous bio-therapeutic agents to the target cells by using different fusion mechanisms and ligand-receptor interactions [3]. Despite these advantages, the existence of natural barriers such as BBB restricts the bilateral transfer of exosomes [4]. This barrier provides an active interface between the blood and brain parenchyma with selective permeability to numerous biomolecules [5]. The barrier type ECs are connected with TJs and hence, limit the paracellular exchange of hydrophilic compounds [6].

Owing to the complexity of BBB structure and selective permeability to exosomes, we endeavor to collect data regarding vehicular traffic through the brain barrier endothelial layer. Understanding the mechanisms of paracellular and transcellular pathways might help researchers to use exosomes as bio-shuttles for the delivery of certain therapeutic agents into the brain [7]. Here, we want to clarify several molecular and cellular mechanisms that differentially regulate the BBB transfer of exosomes through transcytosis and paracellular ways.

## BBB structure

**Fig. 1:**
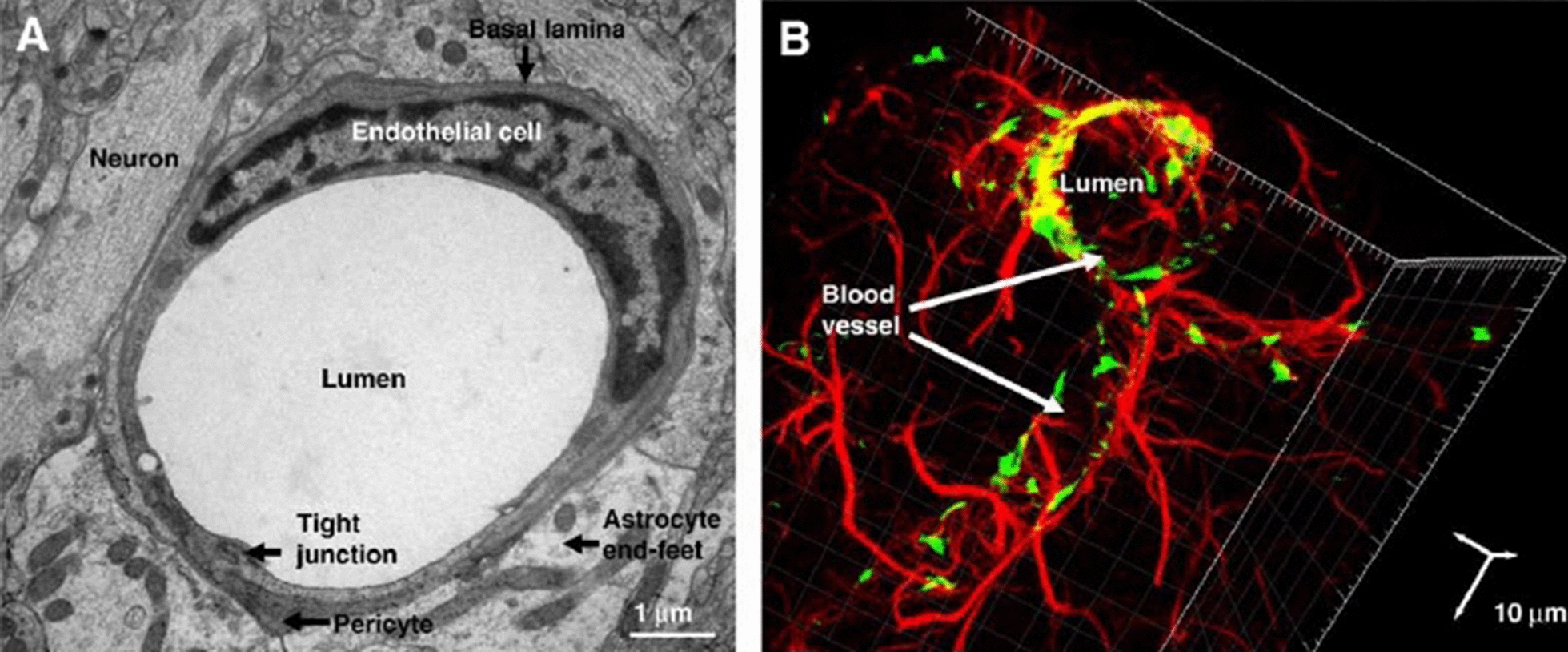
**A** Electron microscopy (TEM) of the neurovascular unit of rat brain section showing the vascular ECs, embedded pericytes in basal lamina and astrocytes, which are close to neurons. **B** Confocal microscopy imaging of cerebral vascular tree in rat brain section, Astrocytes (Red) surround endothelial cells (Green). Vascular ECs and astrocytes were stained for von- Willebrand factor (vWF) and glial fibrillary acidic protein (GFAP), respectively (Adapted with permission [227]. Copyright 2008, Biochimica et Biophysica Acta (BBA)—Biomembranes)

The BBB is known as a cellular interface and a selective barrier between systemic circulation and the brain [8]. This biological barrier is composed of various multicellular structures around the brain vasculature that restrict the passage of larger substances and immune cells from blood circulation to the brain [9]. Regarding the molecular dynamics delivery, there are three different types of barriers between the CNS. They differ in selective permeability and are classified as the BBB mainly constituted by brain microvessels ECs, the BCSFB formed by choroid plexus epithelial cells, and the meningeal barrier formed by arachnoid epithelial cells (Fig. 1) [10].

The BCSFB separates ISF and the CSF from the blood circulation. In the BBB part mainly located at brain microcirculation, the endothelial cells cover the luminal surface of blood microvessels in the cerebral vascular tree [11]. By using precise molecular mechanisms, these barriers maintain the rigorously regulated microenvironment by pushing strict control on passages of various ions and influx of biomolecules to the brain [11, 12]. At the same time, they can simultaneously clear and scavenge the toxins and byproducts away from the brain to the systemic circulation via specialized structures like P-glycoproteins [13, 14].

**Fig. 2:**
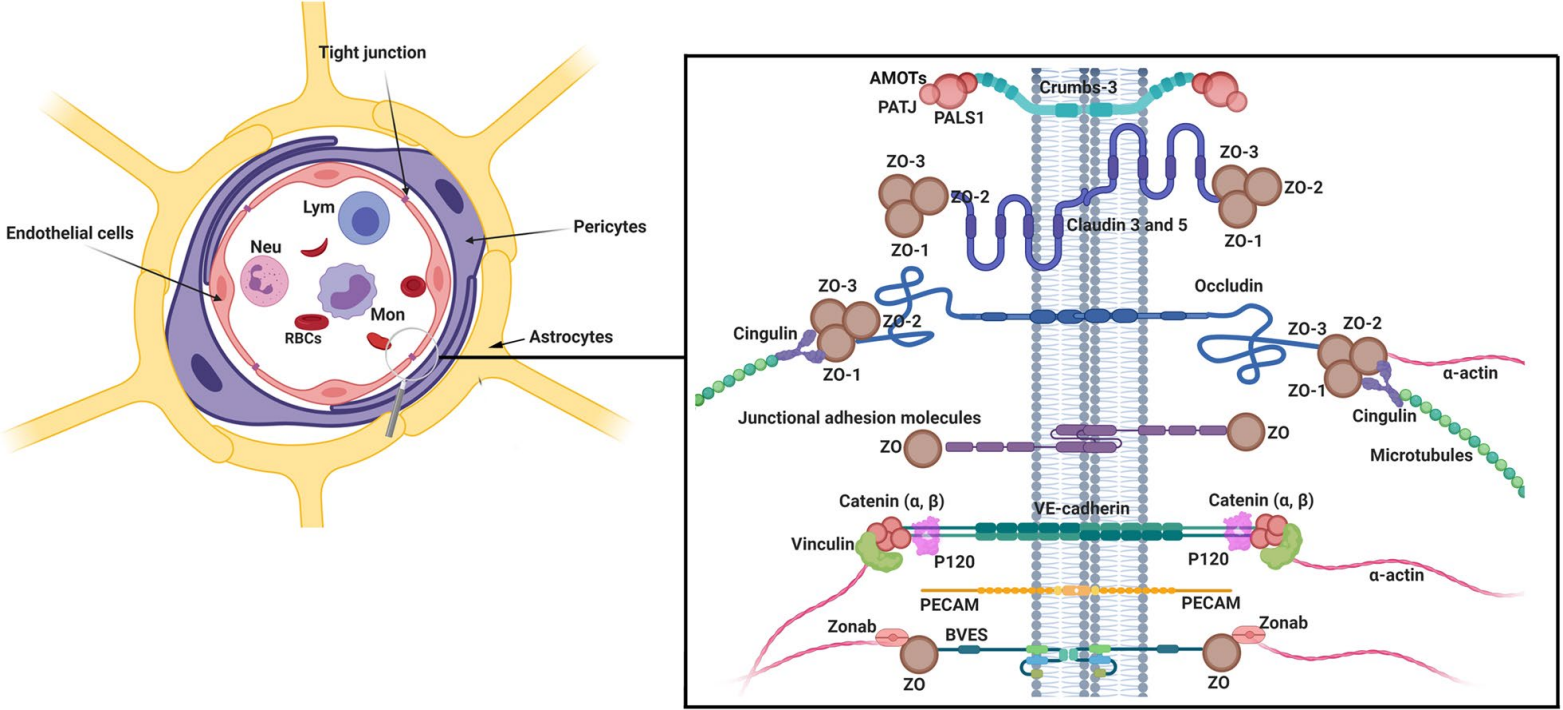
Cellular constituents of BBB neurovascular unit. The cooperation of brain ECs, pericytes, and end-feet astrocytes together establish a unique protective barrier restricting blood cell transfer to brain parenchyma. Pericytes surround ECs throughout the cytoplasm and wrap the abluminal side of ECs. ECs are interconnected by expressing different types of junctional adhesion molecules (JAMs). Claudins, occludin, and JAMs are the most functional proteins present in TJs with active participation in the regulation of various biomolecules exchange. The transmembrane adhesion complexes are linked to the cytoskeleton through a series of cytoplasmic adaptors including zonula occludens (ZO)-1, ZO-2, cingulin, Jacob, membrane-associated guanylate kinase inverted (MAGIs), and Membrane palmitoylated proteins (MPPs). The TJs interact with basal adherens junctions (AJs) and are linked to the actin/vinculin-based cytoskeleton by catenins. *Lym* Lymphocytes, *Neu* Neutrophils, *Mon* Monocytes, *RBC* Red blood cells

The barrier ECs with unique properties like TJs, a limited amount of pinocytic vesicles, presence of the specialized carrier, and transport systems, are some of the core elements of the BBB compartment which recognize and participate in the active control of the passage of biomolecules. The existence of continuous and seamless connections through TJs between neighboring ECs limits the passage of large molecules across the BBB and faces the researchers with major problems in drug delivery [15]. On the other hand, ultrastructural imaging revealed that the intracellular flux is low in ECs due to low transcytotic activity [16]. Therefore, the transfer and passage of biomolecules through the BBB are limited to specific mechanisms [17]. In this regard, distinct receptors and transporters present on the EC membrane regulate the active exchange of the various biomolecules [18]. In recent literature, the term BBB has been widely used to describe the complex structure consisted of neurovascular unit components, including pericytes, basal lamina, astrocytes, microglia, and neurons [19]. The layer creates a dynamic cerebral microenvironment and controls cerebral blood flow (Fig. 2) [20].

ECs of the BBB are front-line cell layer components exposed directly to different biological molecules in circulation. From morphological and functional aspects, ECs are different in the various vascular beds. Microstructural studies have shown that the existence of gate function in the BBB structure is due to TJs and adherens junctions between ECs [21]. Thanks to the presence of such structures, the splits are closed up, and ECs are tightly intertwined, limiting the paracellular permeability and making brain tissue immune privileged. By owning such a unique physical contact, lipids and proteins commute between the basolateral and apical surface of the ECs membrane [22].

To maintain continuity of EC-to-EC contact, proteins like occludin and claudins are the main elements of TJs proteins between juxtaposed ECs [23]. Claudins are known as membrane proteins that contain four transmembrane domains, a cytoplasmic tail, two extracellular loops, and a short amino terminus [24]. The extracellular loops have a critical role in the structural backbone of TJs and the regulation of paracellular ion transport [25]. Of note, claudins 1, 3, 5, and 12 bind to scaffolding proteins, namely ZO-1, -2, and -3 through carboxy-terminal binding domains. Studies have shown that occludin is not as important as claudins in the formation of TJs, but there are structurally similar to claudins and participates in cell adhesion (Fig. 2) [26]. The activity of occludin and claudins could develop high TEER via the promotion of tight and adherens junctions [27]. JAMs are one of the other participating molecules in the integrity of BBB. They regulate leukocyte attachment and migration, paracellular permeability, tensile function via interaction with actin and cytoskeletal proteins through diverse intracellular signaling cascades [28]. JAMs belong to the immunoglobulin superfamily consist of a single transmembrane domain, an amino-terminal domain associated with dimerization, an extracellular domain with two IgG-like loops, and a short cytoplasmic carboxy-terminal tail. The cytoplasmic tail is in close contact with scaffolding proteins such as ZO-1, AF-6, and per-3. It was suggested that cadherins are one of the major proteins that belong to JAMs [29]. As previously shown, cadherins are integrated into cell membrane proteins, and their activities entirely depended on calcium ions. Cadherins participate in cell-to-cell connection via specific interactions, namely homotypic adhesion [30]. For instance, VE-cadherin, which is found in great abundance in BBB endothelia [31], participates in the regulation of endothelial permeability through the up-regulation of claudin-5 [32]. Scaffolding proteins such as ZO-1, -2, and -3 belong to the MAGUK that connects actin to TJs proteins through carboxy-terminus by using multiple PDZ motifs. These proteins can bind to cytoplasmic effectors and signaling proteins via SH3 and guanylate kinase domains [33]. ZO-1, -2, and -3 are in close interaction with adherent junction proteins through α-catenin. In addition to ZO proteins, other scaffolding proteins such as par-3 and -6, afadin-6, actin, cadherin binding proteins, and JAM-A with a single PDZ domain are involved in cell adhesion and polarization. Interestingly, ECs of BBB also contain some PDZ domain free scaffolding proteins like cingulin limit the transfer of large molecules and ions [34].

Pericytes are other constituent members of the neurovascular unit and localized on the abluminal side of ECs. They wrap around ECs with their podia extensions [20]. These cells are located and resided in the basement membrane of precapillary arterioles, capillaries, and postcapillary venules, make connections with endothelial cells through peg–socket-like structures [35] and are actively involved in the exchange process through the BBB by the regulation of gap junctions’ structure. Without any exaggeration, the relationship between pericytes and gap junctions enables these cells to transmit contractile forces to other cells after the recruitment of adhesion plaques [20]. By inducing Mfsd2a in barrier-type ECs, pericytes can regulate the transportation system. To this end, pericytes stimulate polarization in astrocytes and strengthen the attachment of astrocyte endfeet to the ECs [36].

Astrocytes, which belong to the glial cells in CNS, are the mediators between neuronal cells and vasculature systems while controlling the dynamic of CNS signaling pathways. For instance, these cells participate in neurotransmitter, ion, and amino acid homeostasis within the interstitial area of the brain parenchyma. Astrocytes express particular ion transporters (Kir4.1 and Na+/K+-ATPase) for potassium (K+) buffering and recycling of neurotransmitters like glutamate and conversion to glutamine by uptake glutamate via EAAT1 and 2 transporters [37]. It has been proved that mature astrocytes have a specific role in the maintaining of BBB integrity by the up-regulation of the TJs proteins through different signaling pathways [38]. For instance, the sonic hedgehog is activated via GDNF, bFGF, and TGFβ secretion. Different transporters such as P-glycoprotein and glucose transporter 1 are expressed by the astrocytes [39]. Overall, the close cellular and molecular integration of astrocytes are important in maintaining the BBB integrity and function [40, 41].

## Exosome biogenesis and abscission

Various types of cells can communicate with each other in a juxtacrine and paracrine manner. In a paracrine cell-to-cell connection, an array of growth factors and mediators are released to the microenvironment niche via different biological packs [42]. To this end, different nano- and micro-sized structures such as EVs, [43] are categorized based on cellular origin, dimensions, biogenesis, and physicochemical properties [44]. As a common scientific belief, EVs are originated from the plasma membrane and endosomal pathways [45]. As above-mentioned, exosomes are touted one of the important classes of EVs with an endosomal origin and size of about 50–100 nm in diameter. These nano-sized particles have a critical role in cellular homeostasis, intracellular communication, and molecular mechanisms associated with physiological and pathological functions [46]. Transfer of cytoplasmic proteins and lipids and genetic materials like miRNA involved in different signal transduction pathways is a way that allows the donor cells to alter specific signaling pathways in the recipient cells [47]. The process of packing biological products by exosomes is in close association with the endosomal pathway and consecutive intracellular multi-step processes. In the early stage, numerous ILVs are formed inside the MVBs [48]. MVBs are a certain subset of endosomes that harbor membrane-attached ILVs. ILVs are developed by direct budding into the luminal surface of MVBs. Upon fusion of MVBs with the plasma, ILVs are released as exosomes to the out of the cells. In an alternative pathway, MVBs cargo is decomposed via the direct fusion with lysosomes [48]. It should be noted that the MVBs are commonly originated from internal and external sources. The endocytic vesicles of external origin are named early endosomes in the peripheral cytoplasm, which further fuse with lysosomes or mature into later endosomes and MVBs. Trans-Golgi network is conceived as an alternative origin of early endosomes that finally mature to MVBs and secretory ILVs [49].

The ESCRT is the machinery sorting system composed of various protein complexes that has a critical role in exosome formation [50]. ESCRT consists of ESCRT-0, -1, -2, and 3 subsets, as well as specific AAA ATPase Vsp4 complex. The mechanism of ESCRT sorting is based on the identification and sequestration of ubiquitinated peptides to certain domains located at the endosomal membrane-mediated primarily by ubiquitin-binding subunits of ESCRT-0. The combination of ESCRT-0, 1, and 2 interacts with ESCRT-3 promote budding into the luminal surface [50]. Some authorities declared that the number of ESCRT subsets and activation rate may differ in different cell lineages [50]. For example, seven ESCRT proteins have been identified in HeLa cells, and suppression of effectors such as Hrs, TSG101, and STAM1 belong to ESCRT-0, and ESCRT-1 proteins could abort the release of ILVs. On the other hand, the inhibition of ESCRT-3 related factors such as CHMP4C, VPS4B, VTA1, and ALIX increase the abscission of exosomes [50]. ALIX involves in the budding of endosomal membrane and abscission via interaction with syndecan. Interestingly, ALIX silencing can affect the exosome composition rather than abscission, so it seems ALIX can affect the cargo or subtypes of MVBs before secretion. Based on some facts, MVB biogenesis could occur in the absence of an ESCRT machinery system [51]. These data show that ESCRT subsets exhibit a different mechanism of action on exosome biogenesis and secretion [52–54].

**Fig. 3:**
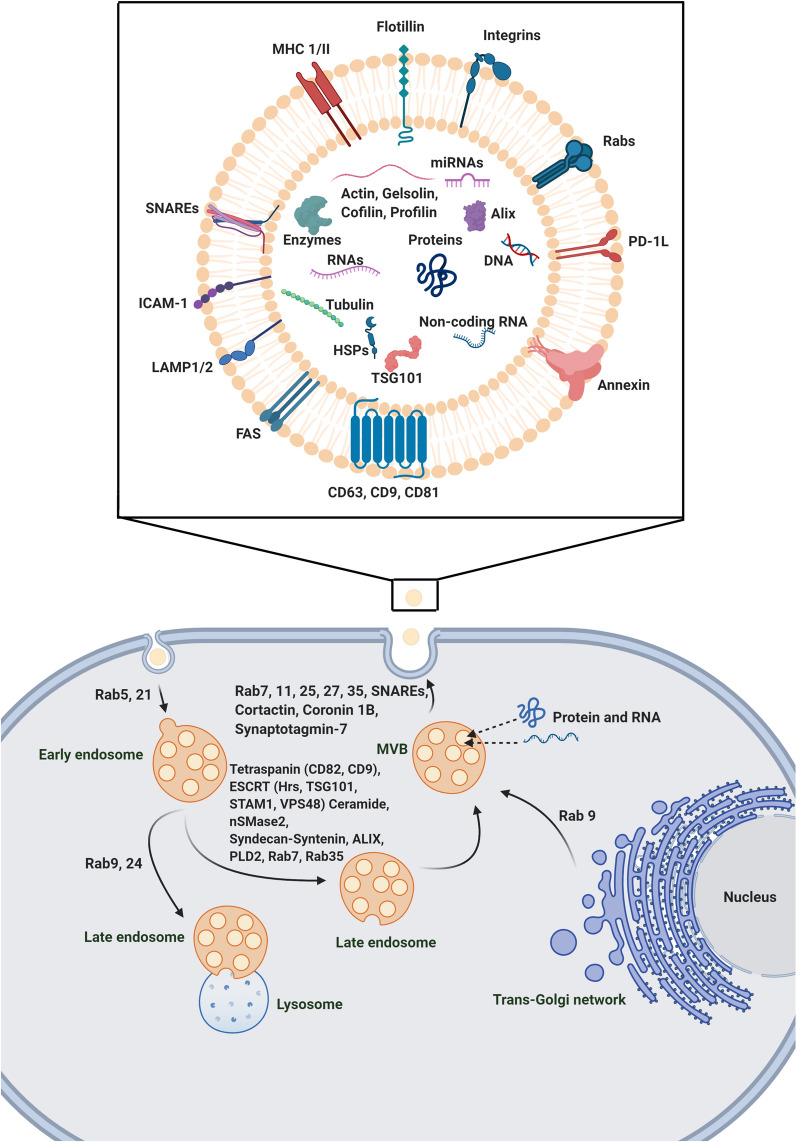
Exosome biogenesis. Exosomes contain different genetic materials, lipids, and cytoplasmic proteins like miRNA, Non-coding RNA, DNA, enzymes, etc. The cargo is transported horizontally among cells. Microvesicular bodies (MVBs) containing intraluminal vesicles (ILVs) are fused with the plasma and release ILVs as exosomes out of cells. Early endosomes originating from external endocytic vesicles or Trans-Golgi network are fused with lysosomes or convert to MVBs. The endosomal sorting complexes required for transport (ESCRT) are composed of ESCRT-0, -1, -2, and 3 subsets, as well as specific AAA ATPase Vsp4 complex, supports budding into the luminal surface. Different Rab proteins are involved in the transportation of endocytic vesicles and early endosomes to integrate with lysosomes or mature to MVBs. Plus, Rabs, Ras, GTPase, together with sensitive factor attachment protein receptors (SNAREs) facilitate the MVBs fusion to the plasma membrane

In a recent study, it has been shown that the ESCRT-independent mechanism sorts exosomal cargo into MVBs via raft-based microdomains enriched in sphingomyelinases. The activity of these microdomains produces ceramides via hydrolytic activity on the phosphocholine moiety [55]. Therefore, the ceramide-dependent pathway has a critical role in the lipid composition of exosomes during biogenesis. Effectors, such as tetraspanins, partake in the biogenesis of exosome and exosomal protein cargo. Microdomains containing Tetraspanins are ubiquitous specialized membrane platforms for the categorization of effectors and receptors in the membranes [56]. The close association of Tetraspanin-enriched microdomains with CD81 contributes to the multi-step sorting of intracellular protein and receptors into the releasing exosomes [57]. The suppression of CD9 that belongs to the Tetraspanin family inhibits the secretion of ILVs in bone marrow dendritic cells mediated by flotillin-1 in the model of the mouse. Other members of the Tetraspanin family have a variety of tasks. For example, the activity of Tetraspanin 8 is not associated with the amount of secreted exosomes, but it can change the sorting and composition of mRNA and proteins toward exosomes. Similar to proteins, lipids participate in the sorting of particular proteins into exosomes [58]. The sorting of certain factors such as CD63, CD81, and flotillin into exosomes is done through inhibition of G protein (Gi)-coupled S1P receptors on MVB’s membranes via the activity of sphingosine-1 [58, 59]. After determining the fate of endosomes, different mechanisms are employed to transport MVBs to the plasma membrane. By direct action of actin, microtubule cytoskeleton, cortactin, which polymerize actin, and tubulin, the transportation, and docking of MVBs are initiated toward the plasma membrane [60]. Oriented transport of exosomes in the cytoskeleton and membrane fusion is carried out via applying the largest family of small GTPase, including Rab GTPase [61]. The fusion of MVBs to the plasma membrane leads to the release of exosomes into the extracellular environment and needs to overcome some energy barriers [54]. To circumvent these barriers, the development of protein–protein and protein-lipid interactions facilitate the MVBs' fusion to the plasma membrane. Rabs, Ras, GTPases, tethering factors, and SNAREs facilitate the MVBs fusion to the plasma membrane. SNARE complex is four coiled-coil helices composed of three or four SNARE subsets (R and Q subsets). Each fusion complex is built up by one R-SNARE and two to three Q-SNARE subsets. The activity of R-SNARE and VAMP7 are critical in the delivery and docking of MVBs to the plasma membrane. Abnormal activity of the N-terminal domain of VAMP7 inhibits the formation of the SNARE complex and accumulates ILVs and MVBs inside the host cells [62]. The superiority and critical role of R-SNARE have been proved in different studies to promote MVBs fusion with the plasma membrane. The inhibition of YKT6 and TSG101 from the R-SNARE family decreases the exosome release. The down-regulation of YKT6 per se contributes to a decrease of TSG101, VPS26/35, and WNT3A factors in the cavity of release exosomes (Fig. 3) [53].

## Transport of exosomes through the BBB

Several physiological transcellular mechanisms are involved in the passage of various substances across the BBB. These include Adsorptive-Mediated Transcytosis, Active Efflux Transport, Carrier-Mediated Transport, and Receptor-Mediated Transport (Transferrin Receptors, Folate Receptors, Lipoprotein receptor-related Protein, Scavenging Receptors, Interleukin-13 Receptor a2, Insulin Receptors, Glutamate Receptors) [63]. ECs are the primary site in the BBB that regulates the exosomal transfer. Upon the physical contact of circulatory exosomes with BBB ECs, some general mechanisms involving in the uptake of EVs such as endocytosis, micropinocytosis, phagocytosis, and plasma membrane fusion are activated to accelerate the inflow of exosomes from the blood into the brain tissue [64].

**Fig. 4:**
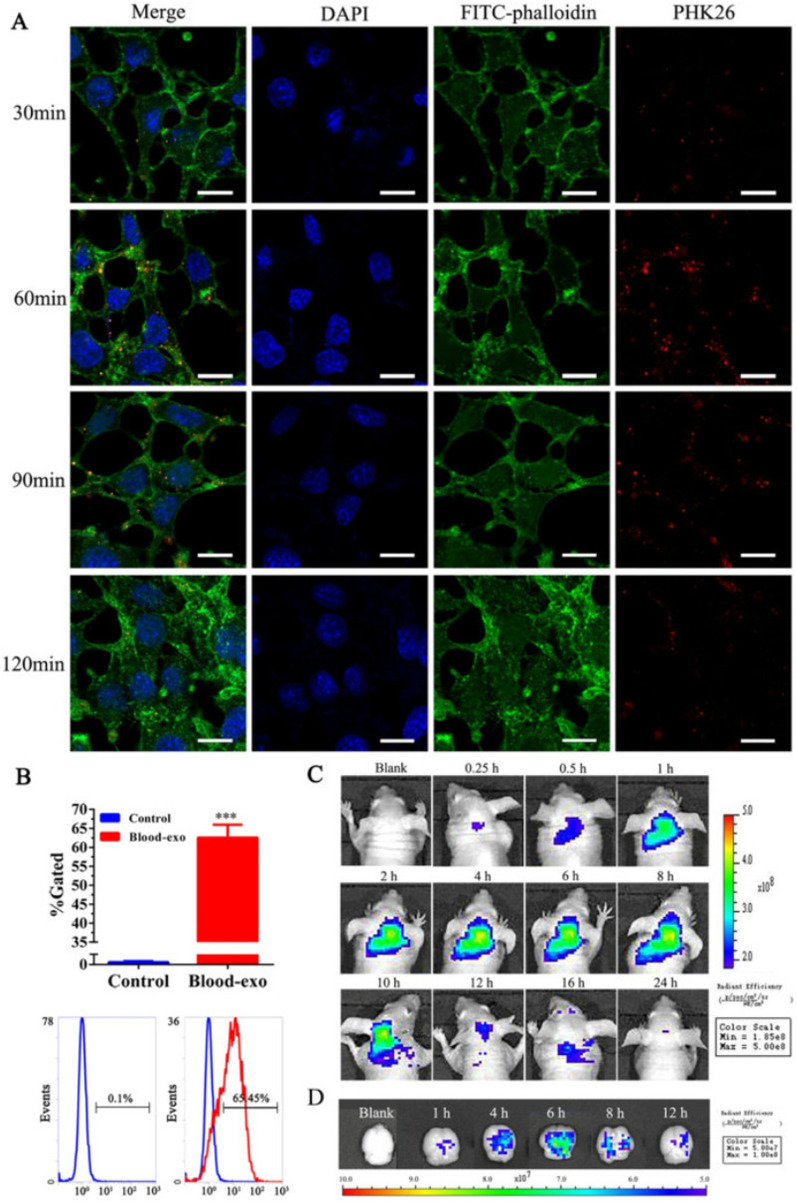
In vitro Internalization and in vivo distribution of blood-isolated exosomes. **A**, **B** Confocal images of internalized PHK26-labeled exosomes by bEnd3 cell lines and flow cytometry analysis in a time-dependent manner. **C**, **D** Representative in vivo and ex vivo fluorescence images that showing the distribution of DiD-labeled blood exosomes after intravenous injection to nude mice [67] (Copyright 2018, Journal of Controlled Release)

In addition to the entry of exogenous exosomes from the circulation into the brain, it has been shown that endogenous brain exosomes are actively budded off and secreted by both glial cells (astrocytes, oligodendrocytes, and microglia) and neurons, showing the existence of both endogenous and exogenous exosomes within the brain tissue [65]. Considering a range of cargo inside exosomes, these nano-carriers could be internalized by barrier-type ECs or different distant cells such as glial cells and neurons [66]. Exosomes labeled with certain fluorescent agents like PHK26 and DiD- are internalized by transcytosis after incubation with brain microvascular bEnd.3 cells. It is postulated that the administrated exogenous exosomes are transferred via brain vascular ECs (Fig. 4) [67]. Determining the exact amount and source of exosomes in the brain may correlate with the physiological state of the cells inside the brain tissues and the integrity of BBB [68]. The entry of exosomes from the circulation into the brain could be summarized in three main mechanisms as follows; physical contact (fusion or ligand-receptor interaction, etc.), paracytosis, and transcytosis [69]. In the fusion of exosomes with the barrier type ECs, exosomes attach and fuse with these cells to release cargo onto the cytosol [70]. Exosome membrane proteins and specific receptors on the ECs surface initiate the existence of gap junction-like communications. In the transcytosis route, there are two possible destinations. Following cellular entry, exosomes are directed to endosomes or degradation. Exosomes inside the endosomes could be transmitted to the abluminal surface of ECs [69].

The molecular mechanisms of transcytosis have not been explained precisely, but some studies have been done regarding the related mechanisms [46]. In a recent study, it was shown that exosome density plays a critical role in the transcytosis of these nano-carriers through the BBB. In support of this notion, the high-density exosome subsets were found in the abluminal side of the in vitro model of BBB [71]. The electron microscopy cleared that the high-density exosomes are smaller (about 70%) compared to the low-density fractions. In contrast, low-density exosomes are accumulated in the luminal side of barrier-type ECs. It seems that the size and density of the exosome are determining factors of exosome transmission from the BBB. Data showed that exosomes passing through the BBB have characteristics similar to exomeres [5]. Exomeres are the subpopulation of EVs with a size smaller than 50 nm that could be isolated from the total exosome population using asymmetric flow field-flow fractionation and simplified ultracentrifugation methods [6]. The biological function of exomeres has not been fully described. Exomeres have an axis role in cell-to-cell communications and carry different cargos into recipient cells to trigger various signaling pathways. There are two subpopulations of exosomes (small- and large-sized exosomes) with different biophysical and molecular aspects compared to the exomeres [72]. It was recently shown that exosome and exomeres have different characteristics in RNA, DNA profiles, proteomic and N-glycosylation patterns [73]. This possibly could be related to having different biological origins, but the exact biogenesis mechanisms of exomeres are still unknown. Further investigations showed that exomeres are involved in the regulation of cell metabolism, such as mTORC1-based pathways [74]. These data suggest the correlation of exomeres intracellular organelles activity such as mitochondria. Due to the smaller size, it is logical to propose that these nanoparticles could cross the BBB interface faster than the exosomes after the loss of BBB under pathological conditions. If we consider the fact that receptor-based transcytosis is the only and significant approach for the delivery of exomeres and exosomes through the BBB, whether the direct interaction and affinity of exomeres and exosomes to the luminal surface of ECs should be elucidated. However, direct evidence for the transfer of exomeres across BBB is still unclear [74, 75]. Noteworthy, proteins like APP, APPL2, and Calsyntenin-1, 2, 3, and BACE-1 are expressed in glioblastoma-isolated exomeres. These proteins participate in the promotion of AD, suggesting the involvement of exomeres in AD pathology. As described already, APP (amyloid precursor protein) trafficking and sialylation by ST6Gal-I enhance the accumulation of amyloid-β peptide inside the brain extracellular matrix [76]. The intricate mechanisms participating in the factor sorting into the exomeres have not been discovered yet. The exchange of exomeres enriched in soluble forms and membrane bounds of T6Gal-I between two sides of BBB reveals a possibly common transfer mechanism for the delivery of both exomeres and exosomes [76]. However, the directionality of transports governed by certain molecular mechanisms remains to be answered. Plus exomeres, exosomes have also been shown to participate in the pathology of AD [77]. Neuritic plaques and neurofibrillary tangles are the major pathologies of AD that formed through the accumulation of Aβ peptides and hyper-phosphorylated tau proteins, respectively [78]. As mentioned above, Alix is a marker for exosomes which has been shown in brain sections of human autopsy tissue along with Aβ plaques in comparison to control groups, proposing the major role of exosomes in the pathology of AD [77]. In addition, an in vitro study indicated the role of exosomes in the transportation of Aβ proteins out of cells followed the propagation of proteins and cleavage from APP [77]. Correspondingly, a study showed the presence of exosomes containing tau phosphorylated proteins in the blood and CSF samples of AD patients [79]. Mutant tau protein has been shown to secrete from microglia by their exosomes to extracellular space in the brain [80]. Based on mentioned data, it seems that exosomes are involved in the transportation of AD-related proteins to the brain by crossing the BBB.

**Fig. 5:**
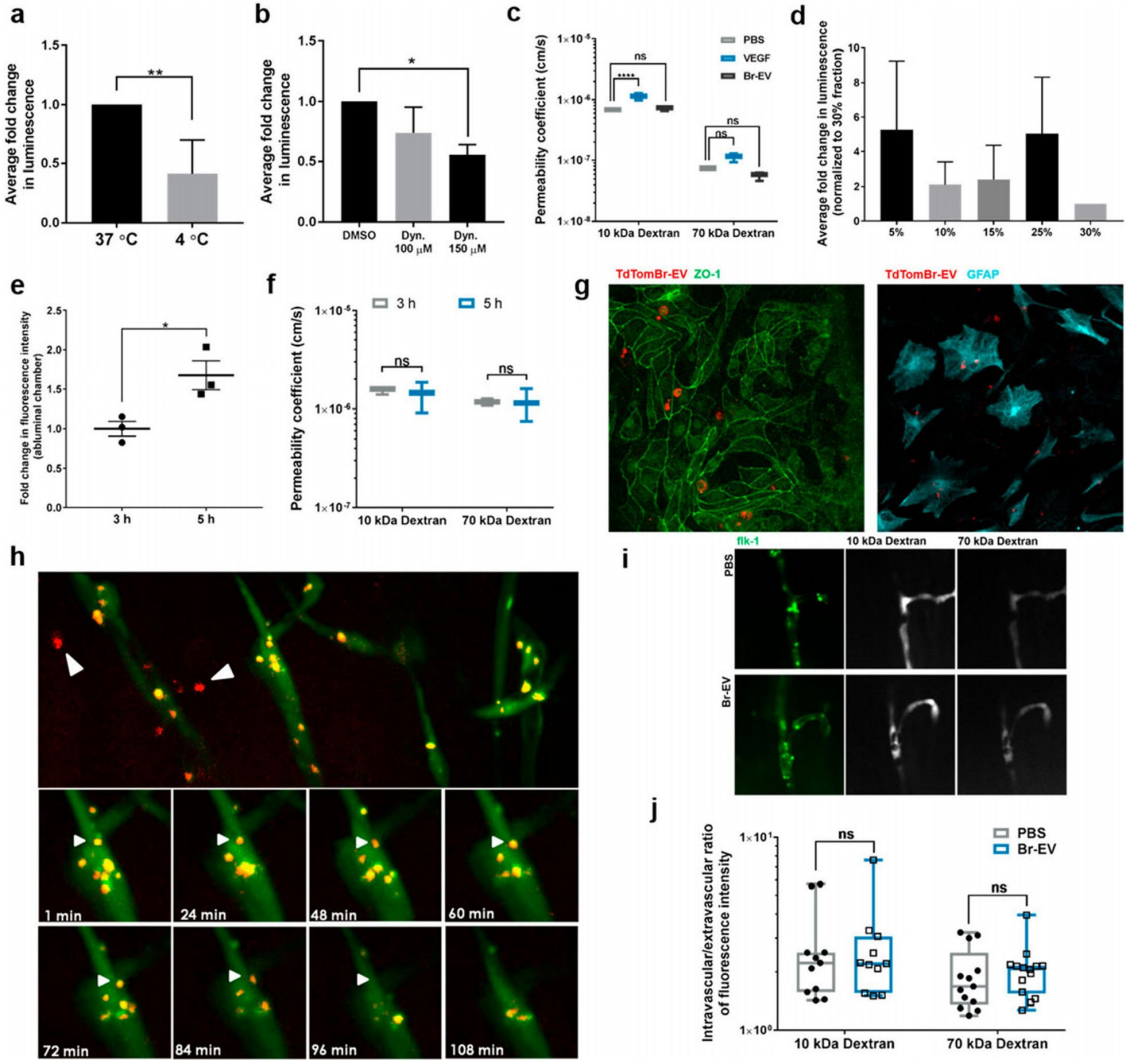
Exosomes conjugated with Br-EVs cross BBB via transcytosis. Changes in immunofluorescence intensity signal under the effects of temperature (**a**) transcytosis inhibition (**b**) and VEGF (**c**) in the abluminal side of the Transwell model of BBB. **d **Average fold change in luminescence intensity of abluminal chamber. **e **The effect of time changes on the fluorescence intensity of crossed Br-EVs to the abluminal side of the BBB-on-a-chip model. **f** Permeability changes of BBB model to 10–70 kDa dextran under the effects of Br- conjugated EVs. **g** Fluorescent imaging of endothelial cells with ZO-1flourescence staining and astrocytes that taking up TdTom-Br-EVs in the BBB-on-a-chip model **h**. Fluorescent imaging of zebrafish brain representing crossed exosomes in the brain parenchyma and the interaction of endocytic vesicles with the abluminal plasma membrane of the brain vascular endothelial cells (white arrows). **i**, **j** Distribution of 10 and 70 KDa of dextran in the zebrafish vasculature system under the effects of Br-EVs in comparison with control (PBS) groups (Adapted with permission [72]. Copyright 2019, ACS Nano)

In a recent study, *Gaussia* luciferase-labeled Br-EVs were incubated with ECs in the luminal side of the Transwell chamber slide by the administration of Dynasore as an inhibitor of endocytosis, which led to a decrease of signal in the abluminal side of the Transwell chamber slide, showing the passage of exosomes through BBB by using transcytosis [64]. Additionally, it has been reported by applying the organ-on-a-chip model of the BBB that, increase in internal cAMP does not play a major role in the context of exosome transport throughout transcytosis [5]. In contrast to in vitro experiments, crossing exosomes through BBB has been shown in zebrafish, which developed mature BBB during 3 days post-fertilization and could be used as a suitable model for BBB. Injected TdTom-Br- EVs in 6–7 post-fertilization have taken up by cells in brain parenchyma. By using live imaging, the movement of endocytotic vesicles has been tracked, which fused with the plasma membrane proposing the transcytosis process [72]. As previously demonstrated, it seems that there is a close association between the transcytosis route and endosome formation [81]. During the process of endothelial transcytosis, the greater number of different particles is directed into the early endosomes, and further, sort out into the late endosomes [82]. In the following, a fraction of late endosomes fuse with lysosomes to degrade the content, and the rest of the endosomal cargo reaches the endothelial plasma membrane at the abluminal surface (Fig. 5) [7].

**Fig. 6:**
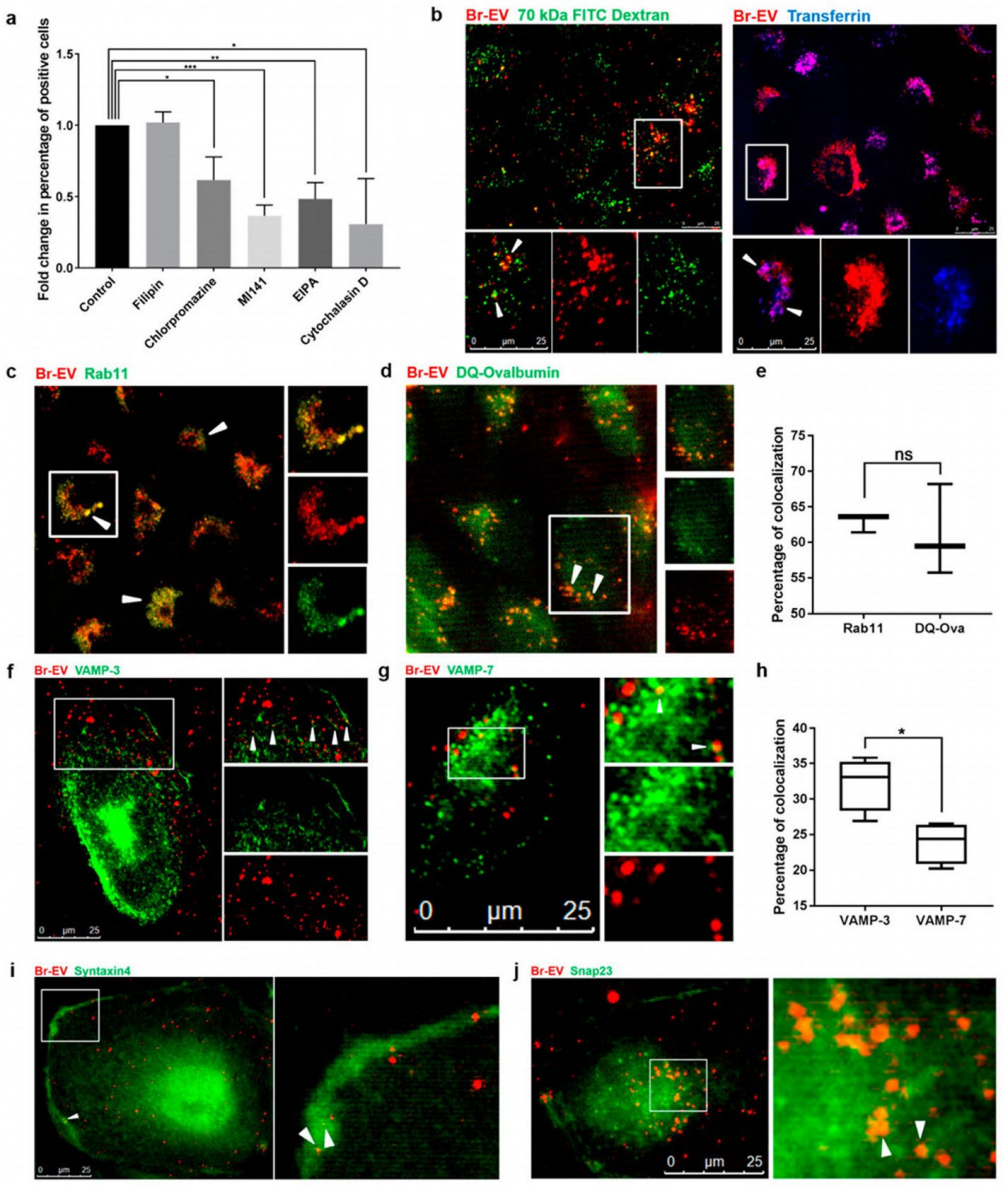
Recruiting recycling endosomes, abluminal SNAREs, and Caveolin-independent pathway by transcytosis-depending of Br-EVs through BBB. **a** Flow cytometry analysis of BBB crossing of Br-conjugated EVs under the administration of different inhibitors of endocytosis pathway compared to the control groups. **b** Fluorescence images exhibiting TdTom-Br-EVs co-localization with dextran and Alexa transferrin, markers of micropinocytosis and clathrin-dependent endocytosis, subsequently. The co-localization of Br-EVs with Rab11 (**c**) DQ-Ovalbumin (**d**) Co-localization of Rab11 and DQ-Ovalbumin positive Br-EVs (**e**) VAMP3 (**f**) VAMP7 (**g**) Co-localization of VAMP-3 and VAMP7 positive Be-EVs (**h**) Syntaxin (**i**) and Snap3 (**j**). The white arrows represent co-localization (Adapted with permission [72]. Copyright 2019, ACS Nano)

To date, several regulators have been detected that orchestrate transcytosis [82]. For instance, the Rab family of small GTPase and Rab recycling endosomes are involved in the transcytosis. On the other hand, as expected EVs are co-localized with early endosomes, which are characterized by Rab5 effector protein [83]. EEA1, an early endosome antigen 1, binds to phosphatidylinositol-3-phosphate throughout the C-terminal domain [84], and plays an axis role in membrane trafficking to distribute the endocytic proteins and leads them to fuse to the membrane proteins [7, 8]. Rab11 recycling endosomes are involved in the transcytosis process by delivering their content to the basolateral membrane [85]. The co-localization of vesicle-containing EVs with Rab11 recycling endosomes could lead them to release of the basolateral membrane [5, 9]. It seems that different endocytotic pathways can act in the sorting of various subpopulations of tumor-derived exosomes to the recycling Rab11 positive endosomes, which have the potential to lead them to recycle, transcytosis, or degradation. The collaboration of Rab11 in late endosomes with VAMP3 soluble NSF attachment protein receptors (Snap23) and syntaxin4 contributes to the release of EVs at the basolateral side. Currently, there is evidence for roles of the SNAREs, vesicle SNAREs (v-SNAREs), and target SNAREs (t-SNAREs), in the fusion of intracellular vesicles with the plasma membrane [10]. Additionally, VMP3 is involved in the exocytosis and recycling of endosomes, but CAMp7 is associated with the fusion of the late endosomes with lysosomes [11]. The data of the same study demonstrated that tumor-derived exosomes co-localized with both of VAMP3, 7. Of note, the co-localization with VAMP7 was lower than VAMP3 with significant differences. These data suggested that the recycling of EVs was a dominant event, and the fusion process in the basolateral membrane has occurred through the VAMP3/ Snap23/syntaxin 4-dependent pathway (Fig. 6) [5, 12].

Macropinocytosis is one of the cell internalizations for EVs, which does not recruit any transportation macromolecules [86]. The Na+/H+ exchange is involved in the macropinocytosis, which acts as a non-specific process by hiring an actin cytoskeleton that leads to the formation of membrane ruffles [87], but this route is limited by BBB flux because of the presences of few pinocytic vesicles in BBB [88]. Additionally, there are clathrin pits on the endothelial surface of brain microvasculature compared to peripheral endothelium, which mediates clathrin-based transcytosis. Following the binding of a ligand to the receptors, clathrin vesicles, which include ligand and receptor together, are formed and deliver their cargos to the opposite side of the cell membrane following endocytosis [89]. Alteration in the activity of some Rab GTPases could facilitate the late endosome fusion with the basolateral membranes [90]. It is worth mentioning that tumor-derived EVs are sorted into transcellular transport by down-regulating the Rab7 [91]. Rab7 is involved in the formation of a ruffle border in the plasma membrane and transfer of early endosomes to late endosomes and then to lysosomes in the endocytic pathway. Additionally, Rac1, as another small GTPase protein, has been shown to co-localize with the GTP-form of Rab7 in the fusion zone. Rab7-Rac1 interaction may associate with the rate of macro-pinocytosis and clathrin-mediated transcytosis because Rac1 plays an axis role in the control of the cytoskeleton, which mediates late endosomal trafficking through microtubules and microfilaments. As previously discussed that tumor-derived EVs with down-regulation of Rab7 can enter to recycling track [72]. The interaction of Rab GTPases, mainly Rab7, with the endocytic sorting system, seems to be critical in the transfer of exosomes through the BBB. It appears that the trans-Golgi network can participate to lead macromolecules back to the basolateral membrane but there is no evidence of interaction of EVs with a marker of the trans-Golgi network (TGN46) [92].

On the other hand, previous studies indicated that exosomes are taken up by neurons via clathrin dynamin-depended endocytosis. The dynamin superfamily is a GTPase family and includes classical dynamins, dynamin-like proteins that are responsible for endocytosis. Besides, oligodendrocytes-derived exosomes communicate with microglia by micro-pinocytosis [93–95]. Of note, exosomal uptake is a selective process. Tetraspanins are a group of superfamily proteins with four transmembrane domains, which interact with integrins as cytosolic proteins. It has been shown that integrins, especially integrin α4, are bona fide of exosomes, suggesting they make a dynamic network with tetraspanins, which leads them to involve in the selectivity process of exosomal uptake [96]. These data indicate that exosome uptake cross over the BBB is based on cell types and exosome contents. Because of its highly selective entity, it seems that BBB resists against exosome transfer via a paracellular route under normal conditions. It's probably no surprise to mention that increased permeability of the BBB is associated with the transfer of exosomes under pathological conditions [97]. Recent data indicated that glioblastoma-derived exosomes enriched with VEGF-A induced the BBB permeability. The increase of VEGF level disrupts BBB integrity by inhibiting the expression of claudin-5 and occludin [97, 98]. In the context of regulation of TJs proteins under different conditions, TNF-α has been shown to stimulate stroke-like conditions in the BBB, which was mimicked in vitro by using Transwell assay. TNF-α significantly down-regulates the VE-cadherin, ZO-1, and claudin-5 expression by shifting them to the cytoplasmic side from membrane [99] and leads to increase EVs trafficking through BBB in vitro*,* suggesting under stroke-like condition EVs can cross BBB throughout the paracellular route. It has not been approved with several experimental data that EVs can cross BBB through the paracellular route in normal conditions [100]. Previous studies indicated that macrophage-derived exosomes internalized into the human brain endothelial cells via various pathways including clathrin/caveolae-mediated endocytosis. In the same study, researchers used filipin as an inhibitor of caveolin-dependent endocytosis, and they did not find any co-localization of EVs with caveolin, proposing no involvement of caveolin-dependent endocytosis on the tumor-derived EVs up taken by BBB endothelial cells [72]. It seems that caveolin/clathrin-dependent endocytosis plays an important role in the initiation of transcytosis of other various macromolecules more than exosomes [72, 101].

**Fig. 7:**
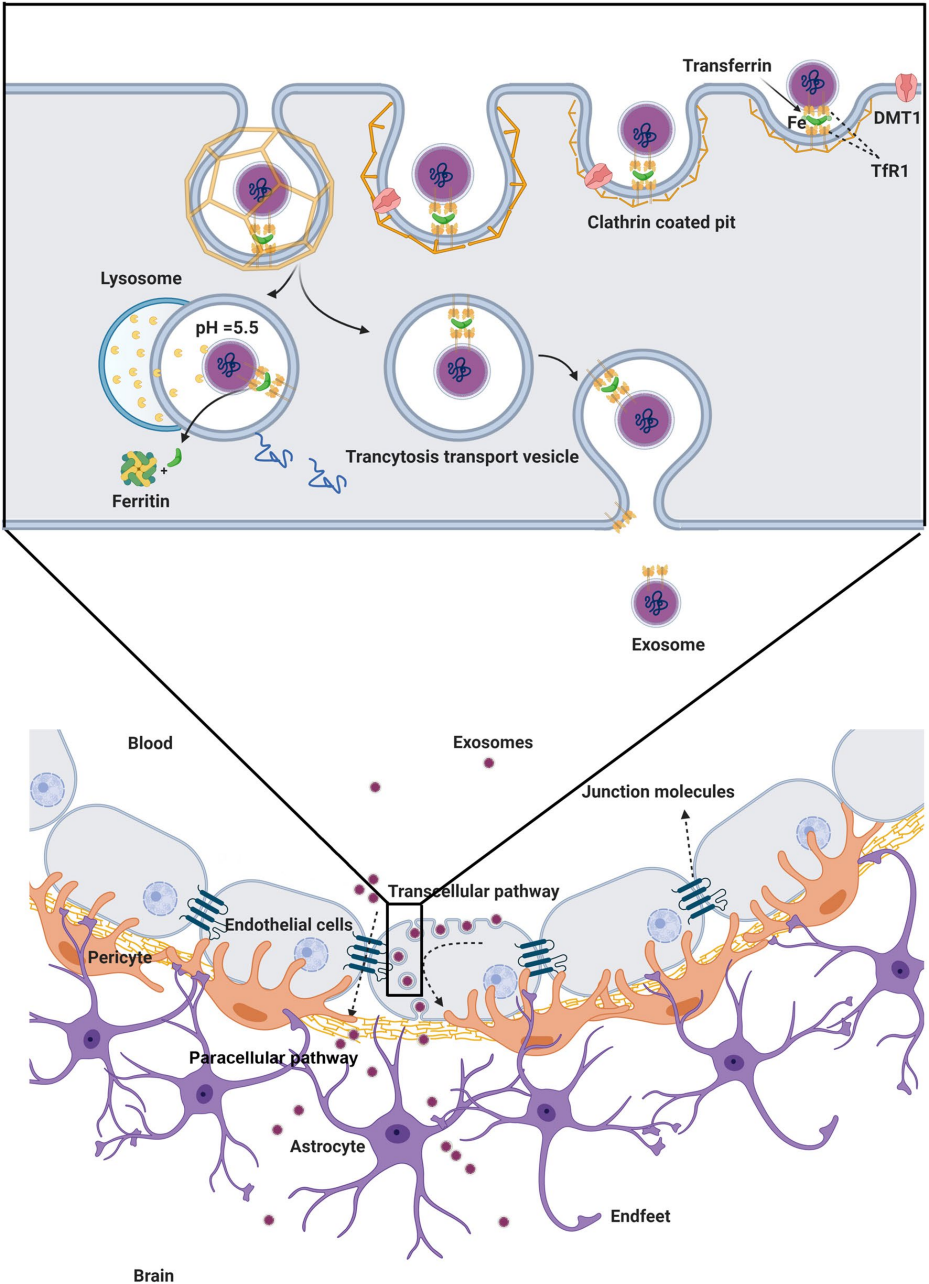
Mechanisms under exosome crossing through the BBB. Exosomes harboring transferrin can bind to the TFR at the surface of brain ECs. In the following step, exosomes are trapped inside the clathrin-coated pit and enter the cells. Clathrin-coated vesicles containing exosomes are transported by cytoskeletal actin filaments and directed to lysosomal or endosomal pathways. Exosomes are degraded throughout the lysosomal pathway inside the cytoplasm, and Fe is released. In the endosomal pathway, exosomes are transferred to the abluminal side of ECs, and TFR is recycled to the cell surface. Exosomes are the potential to pass BBB by recruiting the paracellular route. Under inflammatory and sometimes physiological conditions, exosomes cross BBB by downregulating the expression of TJs proteins and diminishing the TEER. *TFR* Transferrin receptor, *ECs* Endothelial cells, *BBB* Blood–brain barrier; *TEER* Trans-endothelial electrical resistance

As described previously, exosomes interact with brain vascular ECs by recruiting multiple pathways, including caveolin/clathrin-depended endocytosis and macropinocytosis, which were discussed before. Another mechanism of endocytosis is the interaction between LFA-1 and ligand ICAM-1, which are expressed on macrophages-derived exosomes and human brain vascular endothelial cells, respectively [100]. The incubation of macrophages-derived exosomes with LPS-treated hCMEC/D3 as a model of BBB in vitro indicated the interaction of LFA-1 and ICAM-1 in the fusion zone mediates the diapedesis and migration of exosomes across the BBB [102]. Additionally, carbohydrate-binding receptors such as glucosamine-binding C-type lectin receptors, which are express on the hCMEC/D3 cells, are one of the candidates to mediate the internalization of macrophages-derived exosomes into hCMEC/D3 cells. Data showed that LPS administration did not change the interaction between human endothelial cells and exosomes, suggesting that in contrast to ICAM-1, the involvement of c-type lectin receptors in exosome accumulation is independent of inflammatory responses [103]. TfR participates in the exosome delivery mechanism through BBB. It is worth mentioning that blood plasma contains a high amount of transferrin, which mostly binds to TfR on the surface of blood-derived exosomes as well as cerebral vascular endothelial cells. Following this interaction, TfR pinches off the receptor and ligand together, transfers to the cytosolic side of the cell membrane, and leads internalized exosomes to endosomal or lysosomal pathways, which reach exosome to the basolateral side of the cell membrane to cross BBB or degradation pathways, respectively [104]. It has been elucidated by animal experiments that the binding, transport, and delivery of blood-derived exosomes throughout transferrin receptors are mediated by a dynamic endocytosis cycle. In this process, transferrin binds to its receptors on the cell surface and then internalized. In the next step, the transferrin receptor releases its cargo (exosome and transferrin), and then the receptor return to the cell surface to start the next cycle (Fig. 7) [67]. Normally, transferrin receptors are involved in the iron and transferrin delivery in different cell types, including brain capillary ECs. Data showed that an antibody such as OX26, which is used in brain drug delivery, binds to the external domain of transferrin receptors with no interference with transferrin or iron-binding sites. More than 65% of low-weight drugs can pass BBB by this mechanism, suggesting that it is more efficient than other pathways. Understanding the exact mechanisms of TfRs regarding exosome delivery needs more investigations to clarify which domains of TfRs involve in exosome crossing mechanisms [105].

## Selective role of BBB on exosomes transfer during inflammatory conditions

As previously described, exosomes have the potential to cross the BBB during normal conditions. It should not be forgotten to mention the occurrence of inflammatory conditions could disrupt the BBB and increases the exosome transfer as well as permeability? An issue that assumes this hypothesis strongly correlates with the transfer of exosomes from the CSF to the blood soon after the progression of CNS inflammatory changes [106]. The accumulation of exosomes originated from inflammatory neurons or reactive glial cells inside the CNS may alter the integrity and permeability of BBB [107]. The continuous leakage and distribution of these nano-sized particles into the blood not only reflect the inflammatory status of the originating cells but also simultaneously help us to use exosomes as real-time biomarkers for early-stage detection and monitoring the progression of neuroinflammatory status [108]. This was revealed by the generation of large gaps in the BBB compartment at the ultrastructural levels after the occurrence of stroke in different animal models [36]. Of note, exosomes harbor numerous AD inflammatory signals and misfolded proteins, including α-synuclein and prions, which in turn could contribute to the propagation of degenerative injuries and stimulation of adaptive immune response [37]. Preliminary evidence of exosome transfer from the BBB and subsequent uptake by glial cells were presented in the young mice after systemic injection of aged PKH67-labeled exosomes, indicating the modulatory effects of exosomes in the progression of neurodegenerative diseases [36]. Since then, the existence of changes in the levels of hormones, growth factors, and electrolyte misbalances has been addressed in different experiments during the inflammatory conditions with progressive BBB breakdown and CNS-derived exosome infiltration to the blood [109]. After the occurrence of hypoxic/ischemic conditions, a large amount of ionized calcium (Ca2+) is released from the internal stores into the cytoplasm that leads to disruption of BBB via the alteration of junctional proteins [37]. As well as an increase of HIF-1α, VEGF, and nitric oxide in BBB ECs, suggesting the fact that inflammatory conditions could alter the function of proteins and EC-to-EC junction, leading possibly to the reciprocal leakage of exosomes via the paracellular route [38]. Perhaps unsurprisingly, changes in the expression of specific genes, such as miR-212/132, have been documented in another destructive condition that inflammation follows the post-traumatic injury mouse model. The inhibition of BBB TJs proteins mainly ZO-1, claudin-1, and Jam-C by these miRNAs abrogate the integrity of brain microvascular ECs [39]. Whether the transfer of exosomes from blood to CSF or vice versa dominates under inflammatory conditions has been the subject of debate. Similar to inflammatory conditions, the secretion and spread of exosomes originating from cancer cells should not be neglected anymore in the context of BBB integrity. Cancer cell-derived exosomes are rich in cell migration-inducing and hyaluronan-binding protein, which facilitate exosomes spreading through the BBB after attachment to the EC surface and leading to activation of microglia [40]. For example, in a study, the authors proved that mesenchymal derived exosomes decreased TEER of confluent monolayers of bEnd5 cells and passed through paracellular route to the abluminal site in the in vitro condition [43]. Enhanced production of inflammatory cytokines during CNS anaplastic changes, mainly SEMA4D, decreases BBB integrity and increases the possibility of exosome transfer and cancer cell metastasis via the paracellular pathway [41, 42]. Contrary to this claim, it has been shown that the attachment of TNF-α to TNF-receptor on the endothelial luminal surface, resulting in the loss of BBB integrity in pathological conditions and increased the transfer of exosomes isolated from HEK293 cells through the BBB using different transcytosis pathways but not paracellular route [29]. Although studied to a lesser extent, it is logical to hypothesize that alteration of BBB integrity will contribute to the non-selective transfer of diverse macromolecules and even nano-sized particles such as exosomes. Another basic question is whether the increase of permeability to different macromolecules and cell types during abnormal conditions could alter BBB influx or efflux transport systems. As expected, blood is a full pack of EVs shedding from different tissues. It seems that the loss of BBB integrity facilitates the dominant exosome flow from the blood to the CSF through the BBB. The critical role of the paracellular route in the exosome delivery through the BBB is still an aura of ambiguity and needs further studies. The existence of surface charge in BBB limits the transfer of ions and negatively charged particles less than the uncharged or cationic particles. The alteration of EC zeta potential is concurrent with the promotion of inflammation and enhanced permeability [110]. Thus, these changes predispose less or non-specific surface-charge interactions between the EC and negatively charged particles like exosomes compared to the normal condition. Besides, the morphological changes and polarity alteration in astrocytes endfeet at the abluminal surface affect the biochemical and physical connection of the BBB compartment [111]. Therefore, the molecular identity, differentiation expression of certain factors, and alteration of surface charge on the luminal surface could help exosome transfer through the transcytosis along with the paracellular trafficking. The change of net brain entry through the BBB during the inflammation could be touted as a valuable opportunity to deliver EVs alone or loaded with specific factors to the CNS.

## Challenges of exosome-based delivery to the brain

To date, different types of nanoparticles such as liposomes, micelles, PNPs, SLN, dendrimers, nano-emulsions, nanogels, and NLC have been recently used for the transfer of target molecules into the CNS [112]. Liposomes display an appropriate loading capacity for hydrophilic and lipophilic drugs. Despite these advantages, the rapid clearance of liposomes throughout RES (reticuloendothelial system) is the main drawback. In PNPs, drugs are absorbed into polymers, encapsulated, or attached via chemical bonds. Noteworthy, these particles are poorly water-soluble that limits their applications for drug delivery purposes [113]. SLNs have suitable biocompatibility and enhanced physical stability with improved stability of the loading proteins. These particles can release the encapsulated molecules for long times [114, 115]. The prominent crystalline structure of SLNs diminishes the drug loading efficiency and the formulation related to the SLNs can lead to the initial burst release [116, 117]. Moreover, there is an orientation of drug molecules between glycerides or fatty acid chains, so there is a dissolved drug expulsion in SLNs [118]. Hydrogel nanoparticles are nano-sized hydrogels composed of swellable polymer networks cross-linked either by physical or chemical bonds [119]. Nanogels possess high water content, biocompatibility, and flexibility [120, 121]. Unfortunately, the procedure of nanogels synthesis is expensive due to the necessity of solvent removal, the toxicity of remained surfactant, meanwhile weight scaling up and control of mean size is difficult [122]. Dendrimers are in nano-scale dimensions and have star-shaped. The existence of polyvalence features with small size and stable properties make dendrimers appropriate for carrying the drugs [123]. However, dendrimers are directly interacted with cellular components such as proteins, organelles, and membranes, leading to cell lysis [124, 125]. As discussed in detail, the delivery of target molecules by synthetic nanocarriers has different limitations and drawbacks. By contrast, exosomes are used as natural drug delivery systems to transport various types of biological molecule such as genetic materials [126]. The biocompatibility, low immunogenicity, special tissue targeting ligands, stability and long half-life in biological conditions potentiate exosomes as appropriate candidate across different biological barriers [126].

The transfer of various molecules through BBB using exosomes is one of the important strategies aiming to develop novel treatment options for brain pathologies [127]. Nucleic acids can be used for treating different types of human diseases. The major drawback in the field is related to the sufficient delivery of these molecules into targeted cells or tissues. Using various types of vehicles such as viral vectors, liposomal or polymeric nanoparticles, the delivery of nucleic acids is enhanced into the target cells [128, 129]. Exosomes are promising shuttle in the delivery of nucleic-acids (miRNA, siRNA), protein, and small molecules such as curcumin and doxorubicin [130]. The most applied methods for encapsulating different components into the exosomes are transfection, incubation, and electroporation [131, 132]. For instance, Catalase was inserted into the exosomal lumen by using sonication and extrusion, leading to enhanced loading and cellular uptake of exosomes [133]. Likely, exogenous DNA less than 1000 bp can be loaded successfully into the exosomes [134]. To increase the loading capacity, hybrid exosomes were produced to transfect larger plasmids. For this purpose, exosomes were incubated with liposomes and target plasmids for certain time points to enhance exosome-liposome fusion and subsequently encapsulation of plasmids. By contrast, the application of distinct methods such as electroporation yielded low-rate transfection of plasmids into the exosomes [135]. In a phenomenon called CNP biochip, nucleic acid plasmids can be integrated into the exosomes using transient electrical pulses [136, 137]. This system produces large-scale exosomes with large mRNAs from source cells [138]. Along with these modalities, CRISPR-Cas9 technology can be efficient in the introduction mRNAs into the exosomes [97, 139].

As mentioned before, cells can release exosomes in response to physiological and pathological conditions. Therefore, it is logical to hypothesize that the capacity of each cell can differ based on metabolic activity and exogenous stimuli. During past decades, several experiments have been done using exosomes from different sources. For instance, immature dendritic cells are considered a therapeutic cell source for the isolation of exosomes. Dendritic cell exosomes are devoid of surface markers such as CD86, CD40, MHC-I, and -II with low immunogenicity rates. Among biofluids, peripheral blood has large amounts of exosomes originating from different cell types. Moreover, mesenchymal stem cells-derived exosomes are at the center of attention because of their special properties such as immunosuppressive effects after treatment [140, 141]. Cancer cell-derived exosomes are other suitable sources for therapeutic exosomes; they can trigger immune responses mediated by T-cells. Besides, significant tetraspanin content in cancer-derived exosomes makes them eligible to interact with ligands in various types of tissues [96]. Whether these exosomes can be used for normal conditions is the subject of debate. It was suggested that cancer cell-derived exosomes have tropism to the parental cells which limit their availability to non-cancer cells [142]. Plants and fruits are other sources of therapeutic exosomes [143]. It has been shown that exosomes isolated from grapefruit can target inflammatory cancers and present anti-inflammatory effects [144].

As above-mentioned, different inflammatory conditions could disrupt BBB integrity and increase the leakage of different molecules and substances from blood into the brain [145]. On the other hand, counter wise, efflux mechanisms transport CSF content into the blood thus may lead to the reduction of exosomes half-time in CSF [146]. To this end, future studies should address the question of which phenomenon (transcellular or paracellular route) dominates in the transport of substances and exosomes across the BBB under pathological conditions. The apparent specificity of exosomes with cognate receptors on the EC surface suggested that the transcellular route is a suitable approach to deliver exosomes into the brain tissue. Whether both transcellular and paracellular pathways participate in the transfer of exosomes from blood to CSF should not be forgotten. Compared to the non-specific exosome delivery, it is proposed that the modulation of specific intracellular signaling pathways and affinity to the surface receptors accounts for the confident and suitable exosome delivery through the BBB. To be specific, the extent and intensity of BBB leakage are not specified during the pathological injuries.

Besides, attempts must focus on determining the biological fate and direction of endosomal traffic toward transcytosis or lysosomal degradation system inside inflammatory cells near the BBB. The recruitment and presence of different classes of inflammatory cells at the proximity of BBB may disturb the continuous and regular trafficking of exosomes through the BBB [147]. For example, it has been shown that activated macrophages could uptake and degraded exosomal cargo by phagocytosis [148]. This suggests that moderate to markedly active immune cells could limit the exosomes access to the injured target sites [148]. The mentioned challenges should be more investigated regarding brain drug delivery because, in this context, using nano-carriers, especially exosomes may be the more efficient way to deliver therapeutic materials to the brain. As described above, exosomes can pass through the BBB by various mechanisms, but it has been proven that the degradation of exosomes inside the brain ECs is also possible. Previous data proved that exosomes may be directed to the degradation pathways such as the endo-lysosomal pathway and finally degraded by the lysosomes when transfer by receptor-mediated transcytosis and absorptive-mediated transcytosis. It seems that all of the delivered exosomes cannot cross the BBB, and some of them will be degraded by various mechanisms based on the type of exosomes [64, 88]. As aforementioned, some data indicated the fact that under inflammatory conditions, exosomes release their content inside the brain ECs and increased their permeability by changing the regulation of TJs via occludin and claudins [97].

**Fig. 8:**
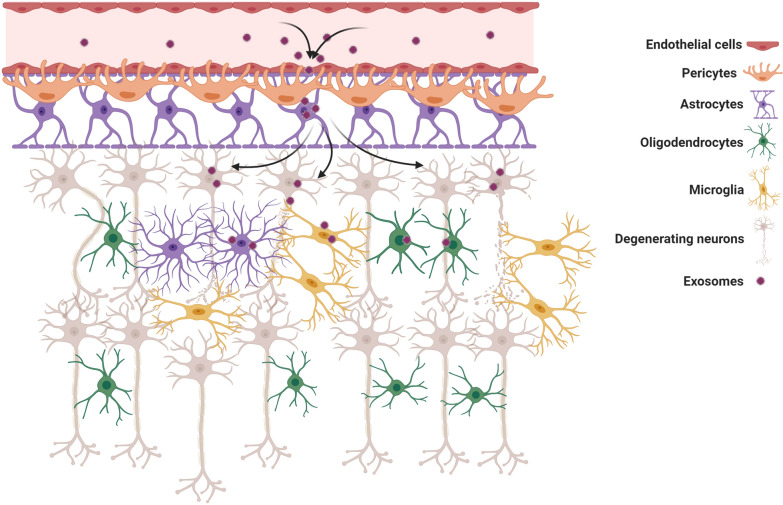
Biological fate and direction of endosomal traffic of exosomes. Endocytic exosomes by receptor-mediated transcytosis could direct to the endo-lysosomal pathway and be degraded by lysosomes inside endothelial cells. Pericytes, astrocytes, microglia, and oligodendrocytes can uptake the exosomes in the abluminal side of the brain. The distribution of exosomes could not be specific to the injured site after crossing BBB. In the context of neurodegenerative diseases, the delivered exosomes may migrate to remote areas

Therefore, the necessary steps that must be taken before exosome drug studies could be as follows:

1. It is mandatory to demonstrate how we can reduce the non-specific distribution of exosomes and how we can direct them specifically to the injured-target sites.
2. Some previous data demonstrated that the pericytes, astrocytes, microglia, and oligodendrocytes can uptake the exosomes [149]. To address this claim, investigations indicated that the EVs, which are produced by glioblastoma cells, are engulfed by astrocytes that stimulate their migration properties [149], but the related mechanisms have been remained unclear. The specific interaction of exosomes with different types of acceptor cells should be defined under pathological conditions to develop better therapeutic strategies.
3. Another imperative challenge that needs to address more thoroughly is the ability of exosomes to migrate to remote sites after passing BBB especially deep brain structures for neurodegenerative diseases. Therefore, in the exosome-based delivery, in vivo trafficking of exosomes must not be neglected because this process limits the transportation of suitable amounts of exosomes to the injured site (Fig. 8).

## Surface modification of exosomes; an efficient strategy to increase BBB cross

As above-mentioned, EVs, in particular, exosomes have the innate ability to cross the BBB [64]. Different research groups reported that a large fraction of systemically injected exosomes quickly is trapped in hepatic, pulmonary, and splenic tissues because of extensive capillary networks and specialized subsets of immune cells with phagocytic receptors [150]. In different experiments, unmodified exosomes can easily spread in biofluids by free dissemination without any targeting capability [151]. Therefore, the targeting ability of exosomes can be amended via using surface manipulation strategies [1]. Conjugation of specific ligands to the surface of transplant exosomes could increase the interaction with the target moieties on cells, while concurrent addition of labeled radioactive, fluorescent, MRI agents is an efficient method for in vivo tracking [152]. Commensurate with these descriptions, it is postulated that surface modification might be a possible approach to increase exosome trafficking through the BBB. Up to date, several modification strategies have been used for exosome delivery. To modify the exosomal surface, different approaches, including aptamer-based surface modification, non-covalent and covalent modifications, multivalent electrostatic interactions, and genetic engineering, have been exploited yet [153].

**Fig. 9:**
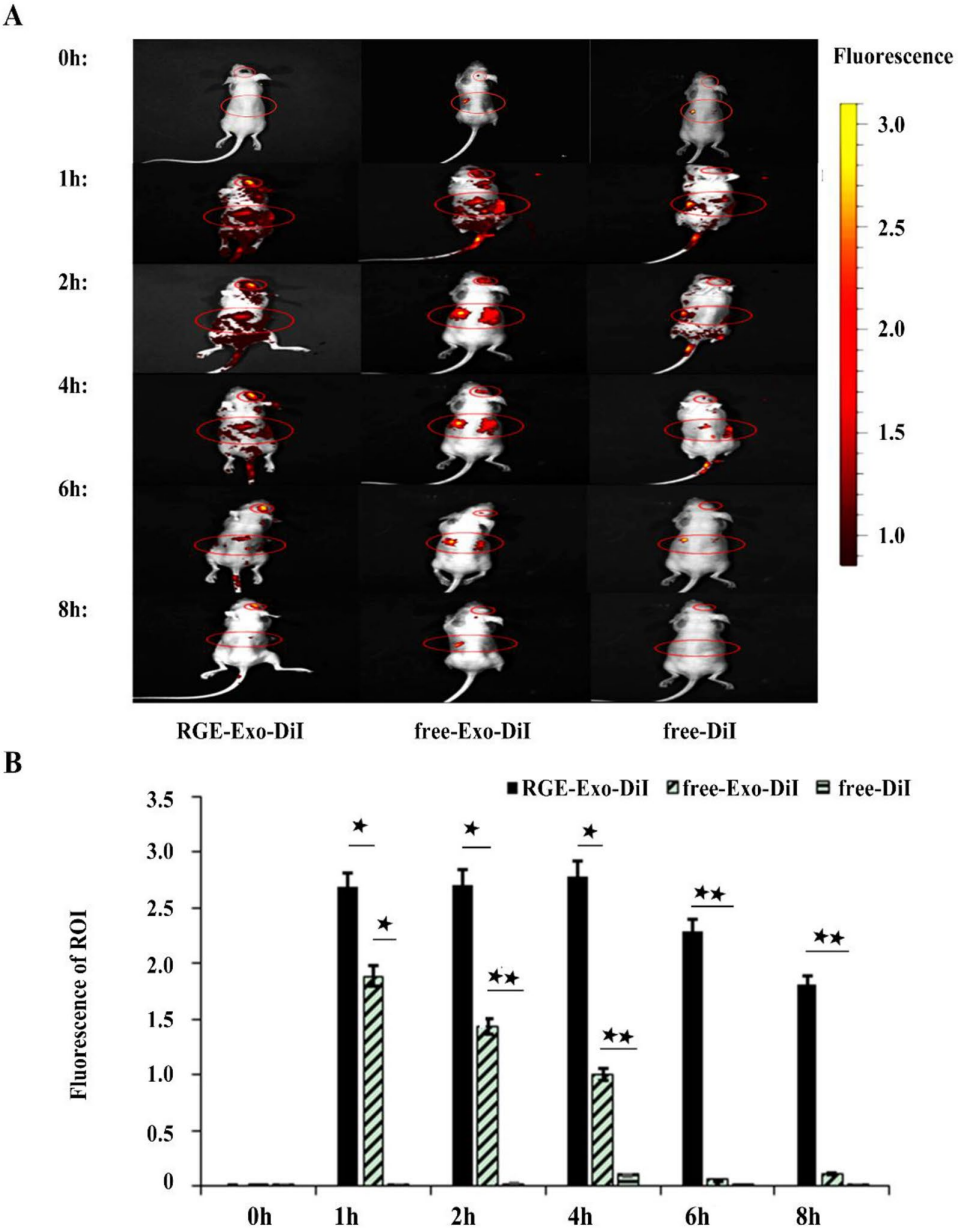
**A**, **B** Time-depended manner of RGE conjugated exosomes with NRP-1 distribution after intravenous injection to tumor-bearing mice and flow cytometry analysis. RGE-Exo shows great ability to target tumor cells [193] (Copyright 2018, Biomaterials)

In click chemistry, for example, the surface of exosomes is functionalized with the application of small, large biomolecules and polymers without affecting exosome function [152]. Chemical modification is a more favorable modality due to ease of synthesis, high throughput, and numerous available chemical reactions [152, 154, 155]. However, the application of several hazardous solvents such as dimethyl sulfoxide, fluctuations in temperature and pressure, and osmotic changes that can disrupt the integrity of exosome structure are inevitable drawbacks related to the traditional chemistries [153]. Click chemistry was introduced in 1999 with great advantageous like mild reaction conditions, easily available reagents [156], using an organic solvent, short reaction time, and most importantly, high efficiency compared to traditional chemical reactions [157–159]. This technique consists of serial chemical reactions to attach the distinct substrates to the biomolecules [103, 152]. Some popular chemical approaches include avidin–biotin complex [160], biorthogonal copper-free click chemistry [161, 162], bifunctional PEG linker [163], and EDC/NHS reaction permit direct attachment of the ligand to the surface of exosomes [164]. Avidin-attached nanoparticles can be coupled by biotin and lectins molecules expressed on the surface of the brain capillary ECs, crossing to the brain parenchyma [165]. Some issues should be considered in this approach before the clinical application like an inadequate strategy for productive enhancement of exosomes without clearly breaking the construction and substance of exosomes used for focused medication conveyance. The biorthogonal copper-free click chemistry, modification of exosomal proteins with alkyne groups are other examples for click chemistry [152]. In the last years, the methods based on bifunctional PEG have attracted intensive attention for the modification of nanoparticles with a wide range of ligands due to their ease of reaction and without the necessity for specific solvents [166]. The principal factor in using these heterobifunctional linkers recognizes the exact functional groups are located on both nanoparticles and ligands. For instance, NHS-PEG-MAL, which is widely used for modification of nanoparticles, easily reacts with amine groups of nanoparticles by its NHS group and reacts with thiolated or Cys-terminated ligands by its functional maleimide groups [167]. Folic acid (FA) is a common small molecule that promotes the internalization of exosomes via endocytosis into the brain because of its receptors overexpression on the BBB [168]. The carboxyl group of the FA can be attached to the surface of exosomes using electrostatic interaction. To date, the EDC/NHS reaction has been used for covalent attachment of FA to the surface of exosomes and other types of nanoparticles [169, 170]. The aptamers are the oligonucleotide sequences (DNA or RNA) that showed promising results in detection and therapeutic purposes. These biomacromolecules have been attached to the surface of exosomes using the EDC/NHS method for remyelination in brain inflammatory disease, especially multiple sclerosis [171]. Smyth and colleagues successfully used copper-catalyzed azide-alkyne cycloaddition in exosomes isolated from mouse 4T1 breast cancer cells [152]. They reported that azide-fluor 545 chemically attached after modification of alkyl groups [152]. Attempts to load different ligands on the exosome surface are based on a variety of exosomal membrane proteins like integrins, Lamp2. Tetraspanins, RAB, actin, cofilin, HSPs, Annexin, etc. are necessary for surface modification [172]. Endothelial uptake of engineered exosomes using receptor antagonists allows passive BBB delivery from the blood to the brain tissue. In genetic engineering procedure, donor cells are forced to secret ligand-bearing exosomes [173]. It has been declared that the efficiency of manipulation occurred in engineered host cells. Besides, this procedure is a significant expense, and most engineered exosomes can’t be easily distinguished from naïve exosomes biofluids. Of available modalities, plasmid vectors (encoding specific ligand) are generally utilized for creating surface-altered exosomes (SMEs). To this end, the coding sequence of the ligand is embedded in the edge between the signal peptide and the N-terminal of the developed peptide of a transmembrane protein. A two-step DNA polymerase chain reaction (PCR) is commonly used by the fusion of a reading cassette into a lentiviral expressing plasmid followed by transfection into the host cells. Eventually, the secreting exosomes can exhibit the specific ligands on their surface [174, 175]. Of note, the possibility of errors in the expression of ligands with high molecular weights, like misfolding and low expression rate, this approach is suggested only for short-length small ligands and homing peptides with 8–20 amino acid residues [176]. For instance, the application of 53-amino acid residues near the C-terminus of the epidermal growth factor is more favorable compared to its precursor format composed of > 1000 amino acid residues [177, 178]. This technology has successfully been utilized for exosome surface functionalization in the phages, bacteria, yeasts, and liposomes [179]. In some circumstances, the surface of the exosome was decorated using the phage display technique [180]. Due to the presence of various native transmembrane proteins in the exosome structure, certain ligands can be over-expressed in the exosome surface using the phage display technique. In this regards, exosomes expressing the Lamp2b-iRGD peptide [181], Lamp2b-T7 [182], RVG, GE11 peptide [183], and hGluc-LA–GFP peptide fusion proteins have been used for delivery of the therapeutic agents (miRNA, DNA aptamer, shRNA, and siRNA) for the alleviation of different brain diseases [182, 184–187]. Despite the advantages of the phage display technique, low repeatability, complexity, expense, and high procedure time restrict its application as a common engineering method. In an experiment conducted by Tian et al., they showed an increased tropism and local accumulation of engineered MSC-derived exosomes loaded with cyclic RGDyK and curcumin into the ischemic brain. The cyclic RGDyK compound was covalently attached to the exosome surface using bio-orthogonal chemistry [188]. Owing to its capacity to bind endothelial integrins, like αvβ3, both linear and cyclic RGDyK have the potential to be used for in vivo application [189]. Enhanced delivery of RGDyK-conjugated exosomes through the BBB occurs via specific attachment to the αvβ3 at the luminal surface. Although αvβ3 involvement has been proved in the BBB transfer of nanoparticles enriched with RGDyK, the data are also congruent with a prominent expression of αvβ3 during pathological conditions [190]. This phase likely leads to enhanced exosome transfer via the specific RGDyK-αvβ3 interaction and non-specific paracellular ways after the disruption of BBB [191]. However, the effectiveness of the RGDyK-αvβ3 system as route delivery to CNS should be interpreted cautiously. Inactivation of α5 and αV subunit using neutralizing antibodies was shown to decrease exosome uptake by ~ 12%, while the inhibition of CD46 reached this value by 39.0% [192]. Considering several types of integrins expressed by ECs in different tissues, one could hypothesize that rapid accumulation of tagged exosomes can occur in highly vascularized tissues such as the liver, lungs once injected intravenously. Also, pharmacokinetic analyses are essential to address the elimination of modified exosomes following injection into the blood. Whether the cellular intensity of integrins in different vascular beds contributes to modified exosome removal, even before BBB cross, is another issue that requires further considerations. The subcellular localization of enriched exosomes with the RGDyK motif and directionality of transcytosis inside ECs needs more studies. NRP1 receptor-mediated delivery of exosomes has been conducted after the conjugation of RGE peptide via click chemistry (Fig. 9) [193]. The exosomes were first loaded with curcumin and iron oxide nanoparticles to suppress glioma U251 cancer cells. It was suggested that such a receptor is a good candidate for BBB transfer of exosomes loaded with iron oxide nanoparticles and other compounds, as indicated by synergistic antitumor effects [193]. Labeling of RGE-exosomes with CellTracker™ CM-DiI dye revealed enhanced accumulation in the tumor regions over time. Being a VEGF co-receptor, NRP1 supports the function of the VEGFR-2 signaling cascade in the BBB ECs [194]. Like αvβ3, activation of NRP1 and VEGFR-2 reduces BBB integrity and mediates the bulk flow of biomolecules into the CSF [194]. Again, whether and how NRP1 receptor-mediated transcytosis could regulate the brain activity of exosomes should be addressed.

The application of a natural receptor on the luminal surface is an alternative thoughtful policy to increase the exosome transcytosis rate. On this basis, LDLRs belong to membrane-bound receptors with a wide variety of functions. These receptors mediate endothelial endocytosis with the ability to attach to different ligands besides lipoprotein metabolism [195]. Unlike nude exosomes, the decoration of exosomes with KLA suppressed the development of human tumoroid U87 cells in vitro and increased extravasation through the BBB, leading to a longer median survival time [196]. Studies at the single-cell level revealed at LDLRs subsets also participate in the direction of endocytic cargo to the lysosomes, and direct data for LDLRs associate endocytosis or transcytosis is absent [197]. Some evidence points to the transcytosis activity of LDLRs subset, namely of LDLR2 [198]. In line with these statements, the development of KLA-loaded exosomes should be done in a way to promote the activity of LDLR subsets that mediate endothelial transcytosis rather than intracellular metabolism and protease activity. Otherwise, it would lead to the elimination of bio-therapeutic molecules at the BBB level before reaching their target site.

As the entry of viruses into CSF and brain parenchyma relies on the binding of viral ligands with cognate receptors on the endothelial surface, the role of viral proteins has been investigated in different studies to deliver distinct cargoes [184]. This sharp-witted approach may possess different advantages like lower toxicity and the lack of bulk manipulation and formulation [199]. In a study conducted by Alvarez-Erviti and co-workers, RVG29, an acetylcholine receptor agonist, was cloned into exosome surface protein Lamp2b in the dendritic cells [184]. Intravenous injection of RVG29-conjugated exosomes with siRNA cargo against GAPDH diminished the expression rate in endothelial, neuronal, and glial lineages [184]. Several issues uncovered by this experiment paved the way for the application of this strategy in brain pathologies that need simultaneous regulation of target genes in different lineages. Despite these advantages, the lack of nonspecific knockdown in multiple cell types displays more improvement over strategies using exosome delivery opsonized with viral ligands. The same protocol was used in the attachment of RVG peptide to MSC-isolated exosomes to regulate pro-inflammatory mediators during Alzheimer’s disease [186]. Unlike nude exosomes, systemic administration of RVG-modified exosomes led to the rapid increase of their number in the brain. The accumulation of MSCs exosomes resulted in statistically significant clearance of Aβ plaques and suppression of pro-inflammatory cytokines TNF-α, IL-β, and IL-6 [186]. These data raise the question of whether cell source and origin may influence neurotropism and flux rate of exosomes through the BBB. The uptake of platelet-derived exosomes by malignant cells and distribution in the tumor niche indicated the attachment of exosomal tetraspanins and integrins with the P-selectin/ P-selectin glycoprotein ligand-1 complex on the surface of target cells [200]. A large amount of Annexin I and V in the cell membrane acts as a receptor for phosphatidylserine on the exosome surface and determines the fate of exosomal cargo in the acceptor cells [201]. High levels of P-selectin and VCAM-1 have been shown at the endothelial surface indicated molecular MRI and micro-sized contrast agents conjugated with monoclonal antibodies against VCAM-1 and P-selectin. Because of their critical roles, data show a moderate and high increase in endothelial levels of VCAM-1 and P-selectin after the initiation of inflammation and neurodegenerative diseases [202]. As noted, these receptors appear to distribute on the luminal surface of BBB ECs, which makes them a possible candidate for the extravasation of exosomes. Importantly, it has been proposed that the activation of VCAM-1 and P-selectin increase platelets function and thrombogenic capacity thereby caution must be taken when developing a target delivery system based on endothelial adhesion molecules [203]. Meanwhile, comparable levels of VCAM-1 and P-selectin are present in the vascular system of different tissues, some of them likely secreted by ECs following inflammation [204]. The broad distribution of VCAM-1 and P-selectin on the luminal surface of the vascular network, along with considerable serum levels of these proteins, raises the possibility of non-specific binding and exosome neutralization before BBB cross.

Direct insertion of the viral genome into the host DNA is another approach to obtain exosomes with equal viral products [205, 206]. In support of this claim, it has been shown that exosomes, also termed vexosomes, harbor different viral proteins from the host cells outside the cells [206, 207]. Such data show the ability of certain viruses to exploit exosome biogenesis and delivery machinery system for intracellular propagation and horizontal transfer [206]. Similarities in exosomal delivery of viral particles dose favor a hypothesis that several mechanisms of exosome uptake could be used by the virions to penetrate host cells. Transfection of cells with the HIV leads to the production of exosomes with prominent CCR5 levels [208]. These data give an important clue that exosomes could be engineered by using viral protein to improve their interaction with brain ECs and increase BBB cross. A Nef is touted as an important factor for HIV entry into the host cells [209]. Early studies investigating the transfection of microglia with a plasmid expressing nef-gfp reported increased Nef exosomal levels and enhanced BBB permeability [209]. Nef has the potency to bind actin filaments connected to occludin, claudins, ZO-1, and JAM proteins [210]. The expression of Nef in brain microvascular ECs reduced ZO-1 and decreased TEER values in an in vitro model [211]. Like covalent modifications, non-covalent modifications such as electrostatic interactions are useful modalities to load fusogenic peptides and cationic lipids on the exosome surface [212].

**Fig. 10:**
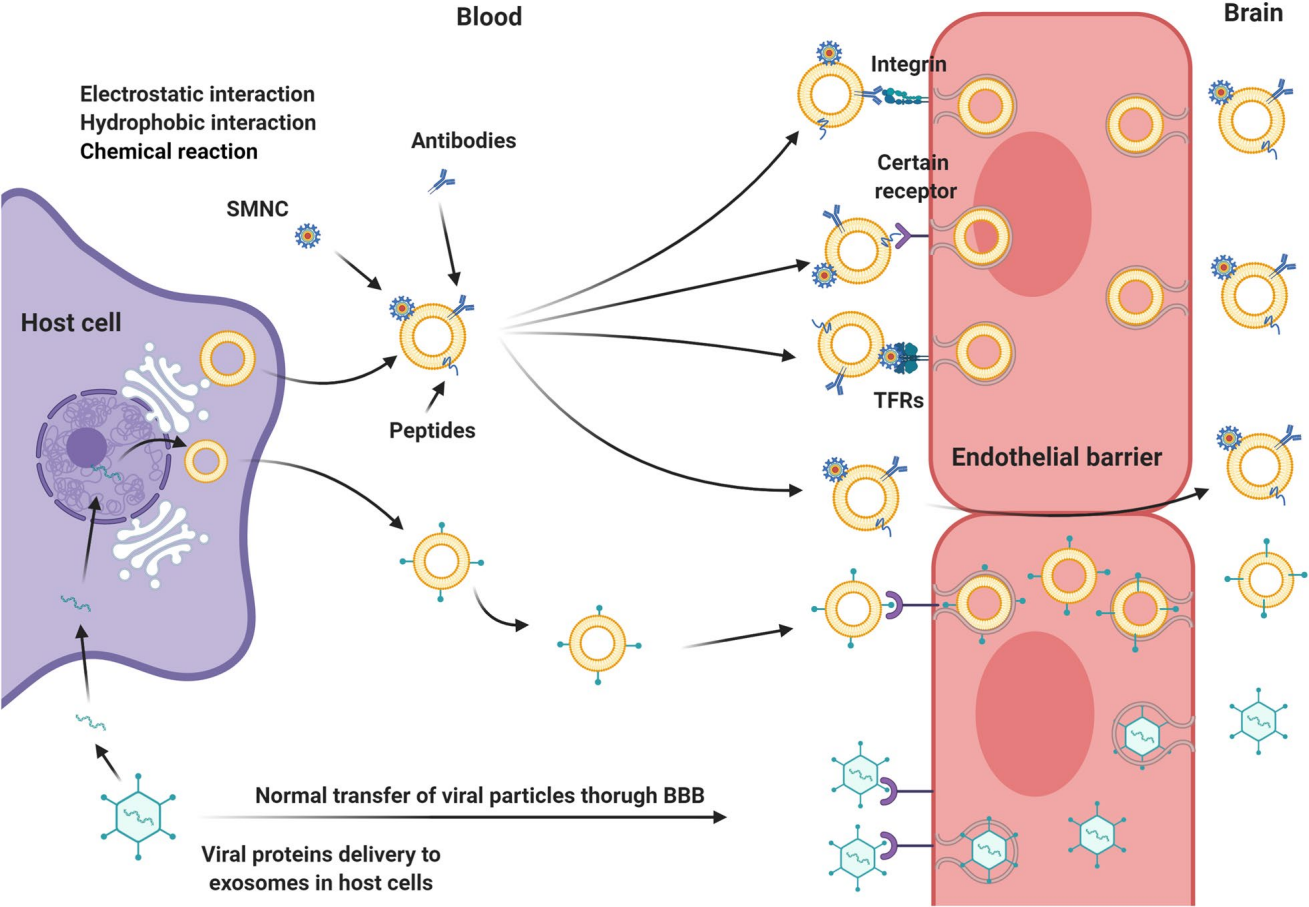
Exosome surface modification strategies. Different surface modification strategies are used to improve exosome delivery to the brain parenchyma. By using click chemistry, RGDyK-conjugated exosomes have been enabled to cross BBB with high intensity through specific binding to αvβ3 integrins on the surface of brain vascular endothelial cells. The capability of RGE surface-modified exosomes is more than nude exosomes to reach the abluminal side of the brain by binding to NRP1 and VEGFR-2 receptors on the luminal surface, which decreases the BBB integrity and increases the transcytosis rate of modified exosomes. Surface engineered exosomes with ApoA-I mimetic peptides (KLA) attach to the low-density lipoprotein membrane-bound receptors (LDLRs) and passage BBB by increased high rate endocytosis or transcytosis pathway. Released exosomes by viral protein transfected cells also show a high capability of BBB crossing. Rabies viral glycoprotein (RVG29)-conjugated exosomes, with specific binding to acetylcholine receptor on the luminal side of brain endothelial cells, cross BBB by highly improved transcytosis pathway. Surface modified exosomes by using CCR5 and Nef (HIV surface proteins) transfected cells acquire a high ability to cross BBB by recruiting transcellular and paracellular routes, respectively. Nef-exosomes decrease the expression of TJs proteins and trans-endothelial electrical resistance (TEER) of BBB

The non-covalent interactions including, electrostatic, hydrophobic, and protein–protein anchoring are the critical features in the modification of nanoparticles that can be applied for exosome functionalization [213]. In a protein–protein interaction system, anchoring CP05 peptide was used because of its high affinity to the exosome surface protein CD63. This system is eligible for targeting of N1ND domain of high mobility group nucleosome-binding protein 1 (HMGN1) in cancer cells [1]. The attachment of RVG peptide to the surface CP05-modified exosomes improved the delivery rate to the brain parenchyma [214]. Along with these strategies, electrostatic interactions have also been employed for targeting negatively charged biological membranes using the positively charged moieties of exosomes. Noteworthy, the cationic pullulans and lipofectamines are the most important positively charged moieties used for electrostatic interactions [215, 216]. Hydrophobic-hydrophobic interaction is another sophisticated applicable for the surface modification of exosomes. Herein, functionalized liposomes with a specific ligand are fused to the exosome membrane following freeze–thaw procedure [217]. The biotechnological approaches are other man-made technologies allowing us to manipulate the structure of natural creatures. The EV ‘cloaking’ platform developed by Antes et al. [218], is the ancestor example of the biotechnological approaches used for the modification of exosomes. This technology supports the creation of methotrexate-loaded EVs functionalized with ApoA-I mimetic peptide as dual-targeting nano-carriers for targeting LDL receptors are located on the BBB and glioblastoma cells. Similarly, the same structure modified by peptide 4F has been prepared for drug delivery to the brain [196]. The surface area modified by SMNC is also another approach used to increase exosomes interaction with the target sites [219] (Fig. 10). In an experiment, the conjugation of transferrin on SMNC-exosomes increased localization into the cancer niche [104]. In contrast to non-BBB endothelium, the BBB interface displays prominent levels of transferrin receptor at the luminal surface, which restrict non-specific bio-distribution of modified exosomes [198]. While surface modification of exosomes is undoubtedly a useful approach to boost the possibility of BBB transfer, the development of modified exosomes with precise targeting with stimuli-responsive capacity needs further studies.

Conjugation of two similar or different nanoparticles is an adequate strategy for the targeting of diseased sites. This novel technology allows merging synthetic exosome expressing glucose transporter 4 with Vesicular stomatitis virus G protein (VSV-G) expressed exosome to creating a pH-responsive construct [220, 221]. Also, the RVG modified exosome/AuNPs hybrid nanoparticles were developed with theranostic susceptibility for brain disorders application [222]. Gene-chem nanocomplexes are a novel hybridization technology that involves the modification of liposomes/polymer particles with various types of targeting moieties for drug delivery to the brain [223]. To overcome some immunological issues related to this technology, the hybridization of exosomes with these synthetic complexes was successfully applied to drug delivery to the brain [224]. REXO-C/ANP/S is a novel gen-chem/exosome nano scavenger which coloaded hydrophilic gene and hydrophobic small-molecule drugs for curing high ROS environment in Parkinson disease (PD) [225, 226].

## Conclusions

In accordance with previous studies and highlighted mechanisms in the current article, exosomes could be considered as intelligent vehicles for drug delivery to the brain parenchyma. Having an insightful view of the recruited mechanisms of exosomes passing through BBB and related challenges could help researchers to ameliorate the exosome-ECs interaction on the surface of BBB and facilitate exosome passage and as results increase the efficacy of the drug delivery to the abluminal side of the BBB.

## Data Availability

Not applicable.

## Abbreviations

APPL2: Adaptor protein, phosphotyrosine interacting with PH domain and leucine zipper 2
AD: Alzheimer’s disease
APP: Amyloid-beta precursor protein
KLA: ApoA-I mimetic peptides
bFGF: Basic fibroblast growth factor
BBB: Blood–brain barrier
BCSFB: Blood-cerebrospinal fluid barrier
ISF: Brain interstitial fluid
CCR5: C-C chemokine receptor type 5
CNP: Cellular nanoporation
CNS: Central nervous system
CSF: Cerebrospinal fluid
EEA1: Early Endosome Antigen 1
ESCRT: Endosomal sorting complexes required for transport
ECs: Endothelial cells
EVs: Extracellular vesicles
GDNF: Glial-derived neurotrophic factor
HSPs: Heat shock proteins
hCMEC/D3: Human brain microvascular endothelial cells
HIV: Human immune deficiency virus
ICAM-1: Intercellular adhesion molecule
ILVs: Intraluminal vesicles
JAMs: Junctional adhesion molecules
LPS: Lipopolysaccharide
LDLRs: Low-density lipoprotein receptors
LFA-1: Lymphocyte function-associated antigen 1
Lamp2: Lysosome-associated membrane protein-2b
Mfsd2a: Major facilitator superfamily domain-containing 2a
MAGUK: Membrane-associated guanylate kinase
MSC: Mesenchymal stem cell
MVBs: Microvesicle bodies
NLC: Nanostructured lipid carriers
Nef: Negative factor
SNAREs: *N*-Ethylmaleimide-sensitive factor attachment protein receptors
NSF: *N*-Ethylmaleimide-sensitive factor
NRP1: Neuropilin-1
PNPs: Polymeric nanoparticles
RVG29: Rabies viral glycoprotein
SLN: Solid lipid nanoparticles
SMNC: Superparamagnetic magnetite colloidal nanocrystal clusters
TJs: Tight junctions
TEER: Trans-endothelial electrical resistance
TfR: Transferrin receptors
TGFβ: Transforming growth factor-β
TNF-α: Tumor necrosis growth factor alpha
VCAM-1: Vascular cell adhesion protein 1
VE-cadherin: Vascular endothelial cadherin
VEGF-A: Vascular endothelial growth factor-A
VEGFR-2: VEGF receptor-2
VAMP7: Vesicle-associated membrane protein 7
BACE-1: β-Site amyloid precursor protein cleaving enzyme 1
